# Understanding neurotropic enteric viruses: routes of infection and mechanisms of attenuation

**DOI:** 10.1007/s00018-024-05450-6

**Published:** 2024-10-04

**Authors:** Valeria Lulla, Adithya Sridhar

**Affiliations:** 1 Division of Virology, Department of Pathology, Addenbrooke’s Hospital, University of Cambridge, Hills Road, Cambridge, CB2 0QQ UK; 2 OrganoVIR Labs, Department of Pediatric Infectious Diseases, Amsterdam UMC, location Academic Medical Center, Amsterdam Institute for Reproduction and Development, University of Amsterdam, Meibergdreef 9, 1100 AZ Amsterdam, The Netherlands; 3 OrganoVIR Labs, Department of Medical Microbiology, Amsterdam UMC, location Academic Medical Center, Amsterdam Institute for Infection and Immunity, University of Amsterdam, Meibergdreef 9, 1100 AZ Amsterdam, The Netherlands; 4 Emma Center for Personalized Medicine, Amsterdam UMC, Amsterdam, The Netherlands

**Keywords:** Enterovirus, Astrovirus, Parechovirus, Picornavirus, Acute flaccid paralysis, Neurovirulence, Virus attenuation, Neurotropism, Gut-brain axis

## Abstract

The intricate connection between the gut and the brain involves multiple routes. Several viral families begin their infection cycle in the intestinal tract. However, amongst the long list of viral intestinal pathogens, picornaviruses, and astroviruses stand out for their ability to transition from the intestinal epithelia to central or peripheral nervous system cells. In immunocompromised, neonates and young children, these viral infections can manifest as severe diseases, such as encephalitis, meningitis, and acute flaccid paralysis. What confers this remarkable plasticity and makes them efficient in infecting cells of the gut and the brain axes? Here, we review the current understanding of the virus infection along the gut-brain axis for some enteric viruses and discuss the molecular mechanisms of their attenuation.

## Introduction

Enteric viral infections, primarily causing acute gastroenteritis, impose major morbidity and mortality worldwide. These infections begin in the intestinal mucosa with invasion of the cells lining the gastrointestinal tract. The control of these pathogens occurs through localized immune responses within the gut mucosa [1]. The first line of defense is the cell-autonomous response (intrinsic immunity) and paracrine signaling to bystander cells, including resident antigen-presenting cells, *via* secreted cytokines and pathogen-associated molecular patterns (innate immunity). This is followed by an adaptive immune response through the gut-associated lymphoid tissue and mesenteric lymph nodes, producing virus-specific antibodies and activating T cells (acquired immunity). This localized control effectively protects against most viral infections while maintaining tolerance to commensal bacteria. However, in rare instances, the initial infection can escape the primary infection site, *i.e.,* the intestine, and spread to other tissues, referred to as secondary infection sites. At these secondary sites, the control mechanisms can be less effective due to increased viral replication and/or overactive immune responses, causing severe complications. This is particularly detrimental during the infection of the peripheral (PNS) and central nervous systems (CNS), often resulting in neurological complications such as meningitis, encephalitis, meningoencephalitis, acute flaccid paralysis (AFP), or death.

Many virus families (*Adenoviridae*, *Anelloviridae*, *Astroviridae*, *Caliciviridae*, *Circoviridae*, *Herpesviridae*, *Picornaviridae*, and *Reoviridae*) are commonly detected in the human gut [2]. Viruses from four of these families use the intestinal mucosa as the primary site of replication. These viruses include astrovirus, enterovirus, hepatovirus, norovirus, parechovirus, rotavirus, and sapovirus. Although numerous viruses and viral families are hosted in the gastrointestinal tract with continued exposure to these pathogens over the human lifetime, only three genera, *Enterovirus*, *Parechovirus* (both from the family *Picornaviridae*), and *Mamastrovirus* (family *Astroviridae*)*,* are associated with distinct CNS and PNS pathology following enteric infection. All these viruses have small positive-sense RNA genomes and are nonenveloped, which can explain the virus stability in the stomach and intestinal environment. The most notorious of these pathogens is poliovirus. Despite decades of research, relatively little is known about the host and viral determinants that drive enteric viruses to the CNS. Other well-known enteric pathogens, such as noro- and rotaviruses, are sometimes associated with CNS/PNS detection and pathologies in human patients [3–5]. However, much less research has been performed on elucidating such infections and underlying mechanisms. Beyond the availability of effective vaccines, a better understanding of the molecular mechanisms directing viral pathogenesis of enteric viruses from the gut to the brain will be crucial in developing new antivirals and vaccines against these debilitating pathogens.

In this review, we provide a general overview of the predominant types of neurotropic enteric viruses, the host cell/tissue complexity along the gut-brain axis and focus on viral spread through several mechanisms to enter the CNS. We summarize possible reasons for the intestinal tract as a selection site and speculate on neuroinvasion and neurotropism determinants with known examples of the molecular mechanisms underlying neuro-attenuation. Finally, we highlight open questions that can stimulate further research on the emergence of enteric pathogens along the gut-brain axis.

## Predominant types of neurotropic enteric viruses

*Enteroviruses*. The initial replication site for human enteroviruses varies between gastrointestinal and respiratory tracts. *Enterovirus alphacoxsackie*, *betacoxsackie,* and *coxsackiepol* species (former *Enterovirus* A, B, and C) are the leading infectious agents associated with enteric and neurotropic diseases, accounting for 70-80% of cases of viral meningitis. Most *Enterovirus deconjuncti* (former *Enterovirus* D) species infect the respiratory tract (EV-D68) or eye (EV-D70), although they can also cause CNS pathologies. EV-D68 is acid-labile and is expected to have limited survival in the gastrointestinal tract [6]. Enteroviruses impact an estimated 30-50 million people annually, with hundreds of thousands being hospitalized.

*Enterovirus alphacoxsackie*. The most prevalent virus resulting in neuropathology is *Enterovirus* A71 (EV-A71), often associated with hand, foot and mouth disease (HFMD). Depending on the virus subgenotype, host age, and immune responses, it can lead to severe neurological outcomes such as meningitis, AFP, encephalitis, and severe systemic disorders [7]. Currently, there has been no established association between a particular EV-A71 genotype and disease severity [8]. In addition, coxsackieviruses CVA2, CVA4, CVA6, CVA10, and CVA16 are increasingly associated with severe cases of CNS infections and are being detected in patients with meningitis, encephalitis, and meningoencephalitis [9–12].

*Enterovirus betacoxsackie*. This group of viruses is associated with the greatest number of sporadic cases of meningitis and AFP, caused by echoviruses E3, E6, E7, E9, E11, E13, E14 E18, E25, and E30 as the most common agents. Coxsackievirus CVB1, CVB2, CVB3, CVB5, and CVA9 infections were also found to be associated with AFP, encephalitis, and meningitis [11–13].

*Enterovirus coxsackiepol*. Poliovirus is the most comprehensively studied enterovirus with neurological involvement. It is mainly found in young children, with 0.5-1% of infected patients developing poliomyelitis. There are three serotypes of poliovirus: PV1, PV2, and PV3 [14]. Other subtypes of *Enterovirus coxsackiepol* neurotropic viruses include CVA1, CVA11, CVA13, CVA17. CVA19, CVA20, CVA21, CVA24, EV-C95, EV-C96, EV-C99, EV-C105, and EV-C109 [11, 15–17]. Although the global campaign has resulted in the successful eradication of polio caused by PV1 and PV3, there are sporadic cases of vaccine-derived polio from PV2 due to reversions or recombinations of oral poliovirus vaccine strains with circulating EV-C strains can result in polio-like disease [18].

*Parechoviruses*. *Parechovirus ahumpari* (HPeV, former *Parechovirus A*), a genus in the *Picornaviridae* family, are highly prevalent viruses (nearly universal seroprevalence for some genotypes) that are primarily asymptomatic or associated with mild symptoms such as fever, rash, gastroenteritis, and respiratory tract symptoms [19]. However, in neonates and young children, they are a significant cause of CNS infections and have been implicated with neurological manifestations and neurodevelopmental delay. Interestingly, severe illness is almost exclusively caused by a single genotype—HPeV-3, although HPeV-4 and HPeV-5 have also been associated with CNS disease [20]. CNS syndromes include AFP, meningitis, encephalitis, and meningoencephalitis [19].

*Astroviruses*. An understudied group of human astroviruses includes classical human astroviruses 1-8 (HAstV1-8) and genetically divergent non-classical VA/HMO and MLB clades. The non-classical genotypes are often associated with cases of encephalitis and meningitis, particularly in the immunocompromised, the elderly, and children [21, 22]. Astrovirus infections, reported to be second to rotaviruses as a cause of childhood viral gastroenteritis, are prevalent among patients hospitalized with diarrhea ranging between 3-10%, depending on the region [23].

*Recent outbreaks*. Regular HFMD outbreaks are caused by EV-A71, CVA6, and other enteroviruses, with the most severe serotypes circulating in the Asia-Pacific region [24]. HPeV-3, the genotype associated with the most severe disease, circulates at biennial cycles and is prevalent globally, with the most recent outbreaks having occurred in the USA during 2022. Several European countries have recently reported cases of severe neonatal sepsis caused by echovirus E11 [25]. There are no approved vaccines or antivirals against this wide range of pathogens, except for poliovirus (globally) [14] and inactivated EV-A71 vaccination in China [26]. However, available EV-A71 vaccines target a single genogroup (C4), with limited evidence for long-term protection against the other genogroups and no protection against other HFMD pathogens (CVA6/10/16) [27].

## How do viruses invade the brain?

The gut-brain axis is a bidirectional network that is significant in maintaining metabolic homeostasis. This axis has garnered tremendous attention in the last decade and has also been implicated in disease. To date, most studies along this axis focus on interactions *via* immune, neural, and endocrine signaling [28]. In particular, the interplay between the gut and its associated microbiome with the brain *via* these indirect routes has been of primary interest (*see box 1*). These routes can also be exploited during viral infections. For example, noroviruses can cause neurological complications through neuroendocrine and/or immune-mediated signaling following intestinal infection [5].

**Fig. 1:**
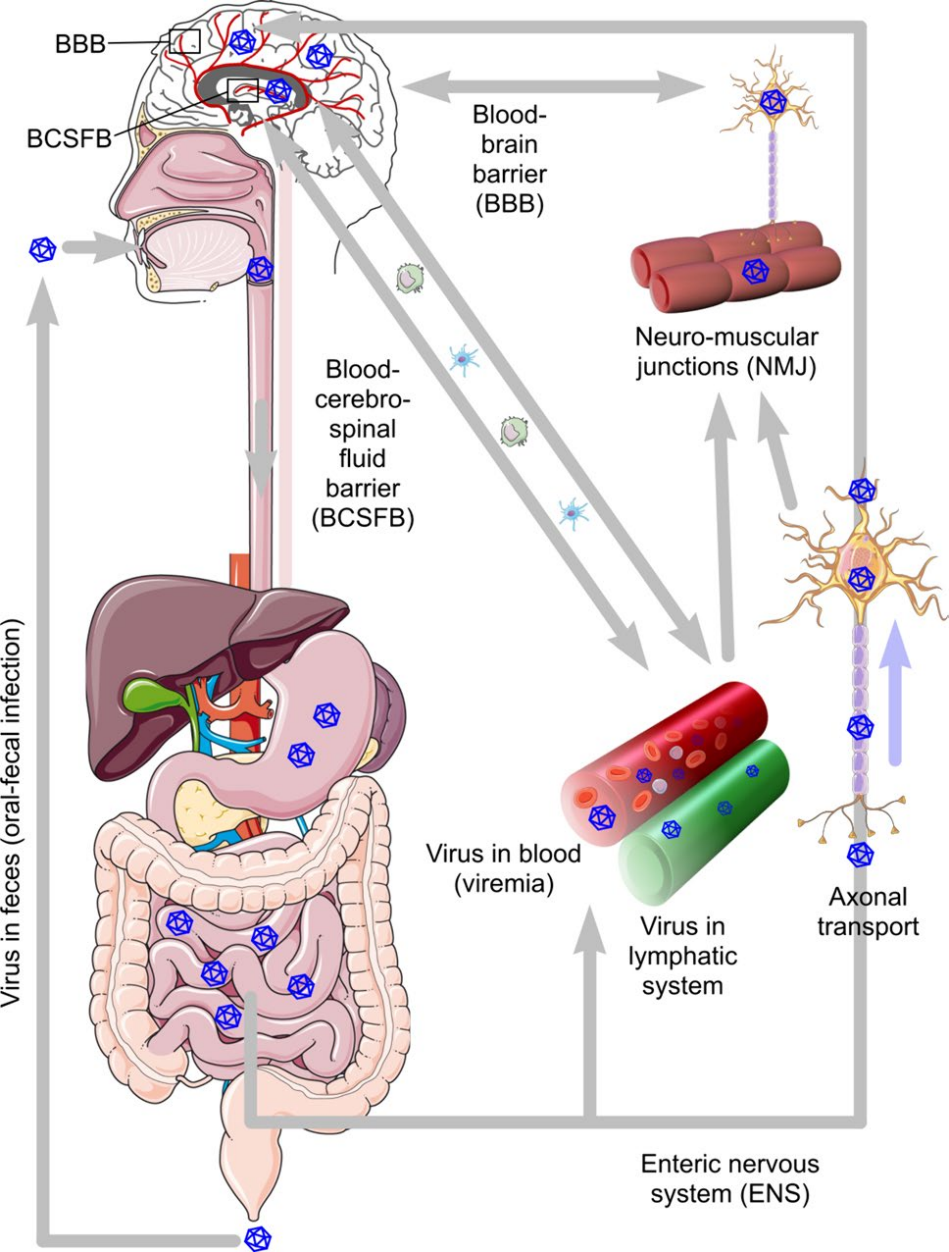
Possible routes of gut-to-brain virus infections. Following primary infection in the intestinal tract, viruses can use the circulatory route (vascular and lymphatic systems) or the enteric nervous system (neuronal route) to reach the CNS. Along the way, the viruses must overcome several barriers before entry and infection of the cells in the CNS. For a full overview of the different cells and the interactions between the different layers, we refer to more comprehensive reviews focused on this topic [29–31]

Given the obligatory parasitic nature of viruses in their interaction with the host, direct infection of the cells in the nervous system will result in more severe consequences for the host. In this regard, the complexity of the gut mucosa provides both bottlenecks and opportunities for adaptation followed by migration to the nervous system (brain, spinal cord, neuromuscular junctions). Once a virus has bypassed intestinal barriers (mucus, epithelial barriers, immune surveillance, *etc.*), two key routes are available for further dissemination into the CNS. The first route is axonal transport along the enteric nervous system (ENS), which is connected to the CNS through the vagus nerve and prevertebral ganglia. The second route of viral dissemination is by fluidic systems (vascular and lymphatic), which link the gut to the CNS through the blood-brain barrier (BBB) and blood-cerebrospinal fluid barrier (BCSFB) (Figure 1).

**Table:** 

Box 1 The gut-microbiome-brain axis
Colonization of the intestine, amongst other sites, by microbes starts at birth and plays a critical role in the continued development and maturation of the intestine [32]. During this critical phase of life, the development of distal sites such as the brain occurs in parallel and is strongly influenced by the colonizing maternal microbiota [33]. Experiments in mice have shown that severe neurological complications arise when their gut microbiome is disrupted [34]. This influence of the intestinal microenvironment on the brain is established and maintained through endocrine, neuronal, and immune signaling [35]
Enteroendocrine cells (EECs) in the intestinal mucosa express over 20 peptides/hormones for signaling and can directly or indirectly interact with luminal components [36]. Molecules secreted by EECs can act on the nervous system by activating receptors on the vagal afferent terminals and ENS. These enteric neurons also respond to signaling molecules such as cytokines, hormones, and neurotransmitters secreted by immune cells in the gut lymphoid tissue [37]. Finally, neurotransmitters such as dopamine and noradrenaline secreted directly by bacteria can act directly on the ENS [38]
Through the integration of these three mechanisms, there is strong evidence to support the significance of the intestinal microbiome for the brain. In particular, the microbiome has been shown to play a role in some neurodegenerative diseases, autism, and depression [33, 39]. As viruses can infect cells responsible for gut-brain communication, it is probable that they have similar (acute) effects on the nervous system following intestinal infection. For instance, herpes simplex virus type 1 (HSV-1) can infect enteric neurons [40], and human norovirus infects EECs [41]. Moreover, the composition of the gut microbiome plays an important role in rotavirus infection and vaccine efficacy [42, 43]. Thus, the indirect role of enteric viral infections on the CNS is understudied and warrants further exploration

Axonal transport of viruses occurs by hijacking intracellular transport mechanisms in neurons. Axons have highly specialized compartments that enable long-distance cargo transport. Microtubules in the axon are used by dynein and kinesins for transport within neurons [44]. Synapses at the periphery of the neurons allow communication between neurons. Once a viral infection is established in a neuron, the virus particles can be transported across the neuronal body *via* the microtubules and passed to a neighboring neuron at the synaptic cleft. Herpes simplex virus type 1 (HSV-1) and rabies virus transport through and between neurons are key examples of viral propagation using this mechanism [45, 46]. In fact, this ability of the two viruses makes them effective tools for transneuronal circuit tracing [47]. However, neurons have defense mechanisms that sense pathogens [48], and HSV-1 spread to the CNS following peripheral infection is still rare [49, 50]. For neurotropic enteric viruses, there is evidence from the poliovirus literature that viral spread can occur by neural dissemination into the CNS following intestinal infection [51]. Whether similar bottlenecks, as seen in herpesviruses, limit invasion of the CNS following infection of peripheral neurons remains to be determined.

Passing epithelial barriers in the intestine also enables access to the vascular (blood) and lymphatic (lymph) systems, acting as portals for secondary infection sites due to their system-wide interconnected networks. Although entry into the lymphatic system does not provide a direct gateway into the CNS, the meningeal lymph nodes are functionally connected by the cerebrospinal fluid (CSF) to the glymphatic system *i.e.,* the waste clearance system of the CNS [52, 53]. There is relatively little known about how viral transport may occur through the lymphatic system into the CNS.

On the other hand, entry into the intestinal vasculature allows systemic circulation and access to the CSF through the BCSFB. The BCSFB is localized within the choroid plexus, producing CSF that surrounds the CNS tissue [54]. The BCSFB consists of a layer of endothelial cells (systemic circulation) in close proximity to a layer of choroid plexus epithelial cells [55]. Tight junctions link these epithelial cells and form a barrier to the CSF [56]. The CSF, produced in the choroid plexus, can be in direct contact with neurons in the CNS [57]. Thus, viruses in systemic circulation can come in contact with the BCSFB and may use similar mechanisms to infect the choroid plexus epithelial cells, as used for infection of intestinal epithelial cells. Following epithelial cell infection, viruses can enter the CSF, where they can potentially infect neurons in the CNS. Neurotropic enterovirus detection in the CSF supports this idea, but it is unclear whether the CSF is a route of entry or exit following initial CNS infection [58].

Alternatively, through the circulatory system, viruses may also encounter the best-characterized brain barrier, the BBB. The BBB is a complex multi-cellular barrier composed of endothelial cells, pericytes, and astroglia [59, 60]. The endothelial cells line the cerebral capillaries and are surrounded by pericytes, with the astrocytic endfeet in close proximity. Unlike the epithelial junctions in the BCSFB, the barrier function of the BBB is governed by the presence of tight and adherent junctions between the brain microvascular endothelial cells. These endothelial cells are also distinct in their lack of fenestrae and tightly control permeability into the brain [61]. Thus, mechanisms used by viruses when infecting epithelial cells may not be directly relevant for bypassing the BBB. Nevertheless, the passage of pathogens through the BBB can occur through three mechanisms: paracellular transport, transcellular transport, and Trojan horse method [62]. Paracellular transport occurs between cells following tight junction disruption due to factors such as systemic inflammation [63]. Transcellular transport can occur through virus transport through the cells either as cargo or due to active infection [64]. Trojan horse mechanisms involve the crossing of infected leukocytes across the BBB [65]. For some viruses, such as EV-A71, there is a correlation between the presence of the virus in the blood (viremia) and clinical severity, suggesting that access to the vascular system plays a role in viral dissemination [66]. At the same time, the fluidic routes provide additional barriers for dissemination, and in particular, antibodies and lymphocytes in the blood can effectively limit disease severity [67]. Some of these protective mechanisms may be absent in immunocompromised individuals, so this could increase the likelihood of neurological complications such as encephalitis [68].

## How can the intestinal mucosa function as a selection site?

As the neurotropic enteroviruses covered in this review exist as quasispecies, the selection of specific variants provides opportunities for further dissemination. Thus, the different routes of CNS invasion described do not exclusively start from the intestine as selection can happen at any site (*e.g.*, respiratory tract), but the intestinal tract’s sheer size offers unique selection opportunities. Regarding surface area, the gut mucosa is calculated to be approximately 32 m^2^ [69]. As a result, it is estimated to house 10-100 trillion microorganisms, including viruses, bacteria, and fungi [70]. These microbes or microbial components may play a role in viral infection or adaptation. For instance, the role of host glycobiology and microbiome is important in rotavirus and norovirus infection of the intestinal tract [71]. Similarly, binding to bacterial lipopolysaccharides, cell membrane component of gram-negative bacteria, enhances the fitness of poliovirus [72]. In addition, mice depleted of their microbiome by antibiotic treatment could not support efficient poliovirus replication in a fecal-oral administration. However, intraperitoneal infection that bypassed the gut resulted in infection comparable to mice with normal microbiome [73, 74]. Usually, astrovirus infection results in mild disease and the absence of the major host- or virus-driven pathology in the gut. However, it was shown that the astrovirus infection causes a substantial decrease in bacterial diversity in infected mice [75, 76]. This suggests that enteric viruses avail the microbiome to promote infection in the intestinal tissues.

The size of the intestinal tract also increases the number of associated host cells that can be involved in CNS dissemination. The human ENS is the most complex unit of the PNS, containing 400–600 million neurons [77]. The intestinal tract is also home to nearly three-quarters of the immune cells in the body [78]. Moreover, the intestine can manipulate systemic blood flow, and the intestinal vasculature receives up to 60% of cardiac output following a meal [79]. The length of the intestinal tract further provides opportunities for pathogen retention and interactions with the peripheral cells involved in CNS dissemination. Thus, these unique features increase the likelihood that pathogens come into contact with cells in the intestinal mucosa functioning as portals to the CNS.

Based on poliovirus studies in mice, the spread of neurotropic picornaviruses from the intestine to the brain is thought to be purely a stochastic process [80]. However, while limiting CNS infection may primarily occur randomly, it is also clear that host and viral genetics are involved, and thus, selection is critical in further dissemination. This is supported by observations of viral [81] and host factors [82] linked with the manifestation of CNS disease or disease severity [82]. Furthermore, the error-prone RNA-dependent RNA polymerase (RdRp) of picornaviruses leads to the generation of viral quasispecies [83]. Once specific mutants, generated due to the error-prone RdRp, overcome the restriction of the initial infection site and cross the bottleneck threshold, they encounter many associated cells, such as enteric neurons, which can lead to CNS infection and related pathologies [84]. As discussed in the next section, a molecular basis exists for such selection events, leading enteric viruses to become neurotropic.

## Molecular basis of neurovirulence and attenuation

**Fig. 2:**
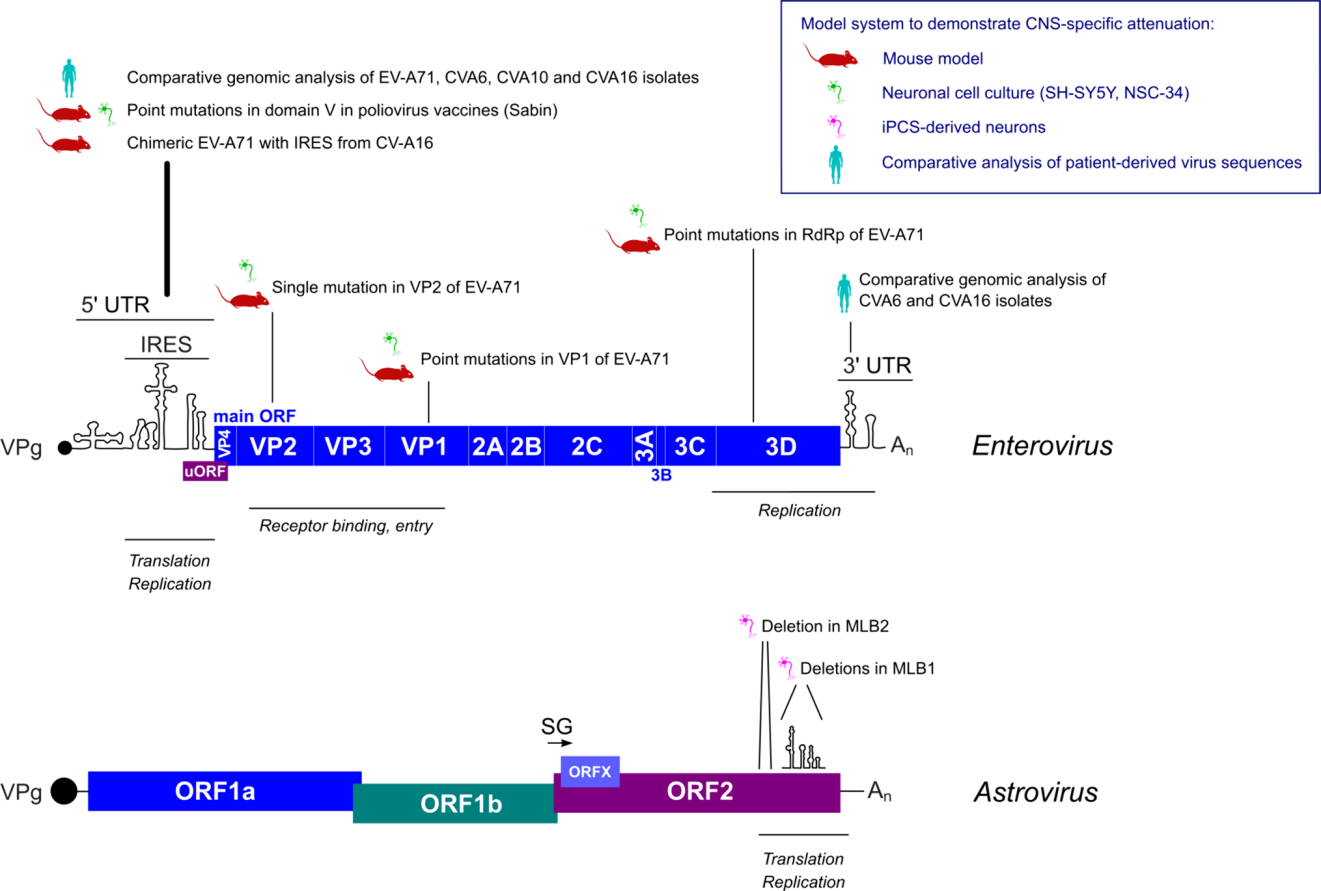
The hotspots in genomes of enteric viruses that are associated with neuro-attenuation. Each example represents a characterized region in the genome of the indicated virus strain associated with virus attenuation in neuronal cells, mouse models, or comparative genomic analyses of isolated viruses. VPg, Viral protein genome-linked; ORF, open reading frame; IRES, internal ribosome entry site; SG, subgenomic promoter; UTR, untranslated region. All references can be found in the main text

The neurotropic enteric viruses discussed in this review section have small (6–8 kilobases) positive-sense RNA genomes that replicate in the cytoplasm of infected cells and belong to *Picornaviridae* (enteroviruses and parechoviruses) and *Astroviridae* (astroviruses) families. The previous studies of the regions in enteric virus genomes provided a range of molecular determinants often specific to a virus serotype or genotype. Genetic manipulation of the RNA virus genomes allows for rescuing recombinant viruses that are attenuated in neuronal infection models but viable in the susceptible cell lines. Such manipulations reveal hotspots in virus genomes that can be attributed to tissue adaptations and extra-intestinal tropism [14, 85, 86]. In addition, such regions can be identified by analyzing multiple patient-derived viral genomes associated with severe and mild cases due to the same virus strain [87]. The viral genomic regions that are linked to the neurotropic properties can be divided into (i) receptor recognition motifs that are usually found in exposed regions of structural proteins, (ii) structured RNA elements involved in translation and replication, and (iii) other host-pathogen interactions including immune responses and modulation of cellular processes like autophagy and vesicular trafficking. The attenuation can be achieved by blocking different stages of the virus life cycle in a cell type-specific manner, limiting the infection at the receptor binding, attachment, entry, translation, or replication stages (Figure 2).

### Internal ribosome entry site (IRES) in enteroviruses

Most neurovirulence determinants in enteroviruses are mapped to the internal ribosome entry site (IRES)—the RNA element responsible for the translation of the enterovirus genome. Translation initiation is tightly regulated in a cell- and tissue-specific manner for both cap- and IRES-dependent mRNAs [88]. Therefore, cell-specific functions are often identified within such regulatory regions. Poliovirus Sabin vaccine strains represent a well-studied example of IRES-dependent attenuation with a single mutation in the IRES responsible for neuro-attenuation in adult mice. Multiple studies have demonstrated the tissue- and cell-specific IRES-dependent translation as a major mechanism behind poliovirus attenuation (reviewed in Racaniello [14]). For example, a single-point C472U mutation in poliovirus type 3 was found to cause a translational defect in neuroblastoma and neuronal cell lines but not in HeLa cells [89, 90]. It was suggested to cause lower replication in the brain and spinal cord [89, 91–94]. However, it was later demonstrated that this attenuation in mice is age and/or immune status-specific [14, 92], suggesting a more complex understanding of neurovirulence determinants that result from multiple host-pathogen encounters during infection and dissemination.

Comparative genomic analyses of strains associated with severe and mild HFMD have revealed clusters of mutations in the 5′ and 3′ UTRs of CVA6, CVA10, and CVA16 that prevailed in genomes found in severe HFMD cases [95]. In addition, similar analyses revealed IRES-specific mutations in neurovirulent EV-A71 strains [87]. A common approach in determining the neurovirulence determinant is based on the distinct neurotropic properties of two closely related strains. Wu and colleagues used two enterovirus strains, enterovirus A71 (EV-A71) and coxsackievirus A16 (CVA16), both of which are common pathogens that cause HFMD but display different neurotropic properties [96]. By exchanging IRES of less neuropathogenic CVA16 in the EV-A71 backbone, the authors observed the abolishment of neuropathological phenotype in mice, suggesting the involvement of IRES in the tissue tropism of EV-A71. Altogether, several independent approaches demonstrated the importance of IRES elements of enteroviruses in neurovirulence. These studies highlight the significance of regulatory RNA elements and translation initiation as a major driver of cell- and tissue-specific pathogenesis. Moreover, since the IRES defines the translation of enterovirus ORFs [97], its regulation between different cell types and tissues can represent the critical determinant of tissue-specific translation, replication, and host responses.

### Structural proteins in enteroviruses

VP1 is a receptor-binding structural protein of picornaviruses, and mutations in this protein can disrupt capsid-receptor interactions. Not surprisingly, such mutations are tightly associated with CNS-related pathologies and cell type specificity that have been extensively studied for EV-A71 [98, 99]. Other examples of neuro-attenuation in enteroviruses include mutations in the structural protein VP2 [100] and combined VP1/VP2-mediated infectivity and lethality in mice [101]. The availability of specific receptors on the host cell surface defines the contribution of receptor binding to neurotropism. In addition, post-binding entry factors may contribute to virion internalization, uncoating, and viral genome release [102]. Together, this makes the structural proteins the critical entry checkpoint in the neuro-invasion.

### RNA dependent RNA polymerase (RdRp) in enteroviruses

The high mutation rate of viral RdRp is key to cell-specific adaptation, virus spread, and eventually, virulence [83]. Reduced mutation rates and recombination frequency can be achieved through mutations in RdRp, discovered by passaging the Sabin2 strain in the presence of ribavirin, followed by sequencing and validation of selected mutations. The reduced mutation rate leads to attenuation of the virus in mice but does not impair virus growth in susceptible HeLa cells [103–106]. In addition, recombination was demonstrated to be a common mechanism to gain virulence via switching IRES and other elements in Sabin2 vaccine [107, 108] and EV-A71 outbreak strains [99] with other circulating enterovirus strains. The speed and fidelity of RdRp are considered significant determinants of recombination frequency [109, 110], which can be manipulated in vaccine candidates by increasing the fidelity of RdRp and/or modifying recombination-prone areas in the virus genomes [105].

### Interactions with host proteins

A growing body of evidence also suggests the active involvement of CNS-specific host factors that modulate neurotropism and viral replication. Virus-specific interaction partners can directly orchestrate this process. Interestingly, intermediate filament-forming peripherin protein (PRPH) was found to facilitate neurotropic EV-A71 entry and replication in neuronal cells, whereas the less neuropathogenic CVA16 was not affected [111]. Specific to neural cells, the RNA-binding protein hnRNP A3 is involved in RNA transport and modification, and its knockdown promotes the replication of EV-A71, presumably through interaction with the IRES and interfering with IRES-dependent translation [112]. Due to high expression and phase-specific regulation of hnRNP A3 in human neural progenitor cells, controlling cell cycle arrest can promote virus infection, which was demonstrated for CVB3 [113]. Regulation and subversion of autophagy is another CNS-specific example, where EV-A71 infection shows no apoptosis or cytopathic effect yet leads to productive virus spread via non-lytic release [114, 115].

### Attenuation in astroviruses

Recently, we described a different example of the RNA structure involved in the attenuation of neuronal infection. Using directed evolution of the neurotropic MLB1 astrovirus strain, we identified a set of deletions in the region overlapping two virus elements: structured RNA elements and cleaved part of the structural polyprotein. Such deletions have enhanced virus replication in susceptible cell lines (Huh7.5.1 and HEK293T) but were attenuated in iPSC-derived neurons. In contrast, the attenuation of closely related MLB2 astrovirus resulted in smaller deletion in the region that was not associated with predicted RNA structures and most likely driven by the yet-to-be-characterized function of cleaved C-terminal part of the capsid polyprotein that is not a part of the virion particle [85]. The full neurotropic potential in these and other astrovirus strains needs further investigation.

### Attenuation in parechoviruses

Much less is known about neurotropic determinants in parechoviruses. However, recent advances in cell culture and organoid infection models demonstrated that the host innate response, not the neuroinfectivity, represents a major driver of neurovirulence associated with the HPeV-3 genotype [116].

Together, these strategies demonstrate the remarkable plasticity of CNS-specific signatures in virus genomes that participate in various stages of the virus life cycle (Fig. 2). Finding similarities in the attenuation of closely related viruses (e.g. IRES-containing picornaviruses) or similar virus-host interactions for a wider range of viruses can pave the way for CNS-specific antiviral therapies. Combining several attenuating mutations is a widely used strategy for attenuated vaccine development [105]. Moreover, these mutations can be transferred to closely related viruses, producing promising vaccine candidates [117]. The involvement of host factors further expands this repertoire and facilitates future research into the host-pathogen interface in the context of CNS infections. It should be mentioned, however, that many neuro-attenuated viruses could also be attenuated in other immune-competent organs, including the gut epithelial and other infected tissues. This depends on the virus attenuation method. For example, passaging in susceptible cell lines and immune-deficient infection models could result in the virus’s inability to replicate in immune-competent cells. So far, neurotropic determinants are not defined for parecho-, noro-/sapo- and other intestinal viruses. Combined, known neuro-attenuation strategies represent a powerful platform for the design of the next-generation vaccine candidates [105] and CNS-specific therapeutics.

## Model systems to study gut-brain transition

The obligatory intracellular parasitism of viruses necessitates using live model systems to understand viral pathogenesis. However, cell lines, although cheap and accessible, are not suited for recapitulating the diversity of interactions driving viral dissemination along the gut-brain axis. The use of complex model systems is warranted to further our knowledge of host and viral determinants governing transport along the gut-brain axis for enteric viruses.

### Animal models

Vertebrate animal models capture the necessary physiological complexity to address questions related to inter-organ viral dissemination/transport. The phylogenetic proximity and physiological similarity to humans position nonhuman primates as important models for studying neurotropic enteroviruses. However, studies in nonhuman primates are expensive and impractical to manipulate for investigating specific mechanisms [118, 119], although they served well to characterize pathogenicity and vaccine efficacy in many studies [117, 120]. As a result, mouse models (wild type, transgenic, and knockout) remain the primary vertebrate models used in picornavirus research [121]. As both natural picornavirus and astrovirus infections are order-restricted, identification of viral entry receptors enables the use of transgenic murine models [122]. However, viral receptors are yet to be identified for many viruses covered in this review. Moreover, the interferon response in mice is thought to inhibit oral infection and restricts studies along the entirety of the gut-brain axis in an immune-competent model [121, 123]. For specific viruses, such as EV-A71 and PV, immune-competent mouse models mimicking natural infection routes have been developed [124]. There are indications that point mutations in the VP1 of EV-A71 may result in differential tropism between mice and humans [125, 126]. Moreover, the identification of (human) host-specific factors will not be possible using animal models.

### Organoids and organotypic models

The use of stem cell-derived organoids and organotypic models has been growing over the last decade in all biomedical research, including infectious diseases [127, 128]. The key advantage of using organoid models is the ability to study viral infection in the host-specific (human) genetic context and the possibility of studying donor-to-donor genetic diversity. These models were applied to study severe acute respiratory syndrome coronavirus 2 (SARS-CoV-2) and contributed substantially to disease modeling during the pandemic [129].

We have extensively used primary cell cultures and organotypic models for studying infection and molecular mechanisms of neurotropic enteroviruses in the cells along the gut-brain axis [97, 116, 126, 130–133]. Using an intestinal enteroid model, we demonstrated the importance of a previously unknown open reading frame for poliovirus infection [97]. We have also characterized HPeV genotype-specific infectivity in organotypic models of both the primary and secondary sites of infection. Our results using these human organotypic models indicate that the intestine, but not the airway, might be the selection site due to a differential cell tropism of HPeV-1 and HPeV-3 [132, 133]. Similarly, a case report of an immunocompromised patient with EV-A71 infection found a mixed population in the intestinal tract but not the respiratory tract, including a neurotropic mutation (VP1 L97R) [134]. More recently, we were also able to show using neural organoids that genotype-specific neurological disease association of HPeV could be due to differences in (exacerbated) inflammatory response rather than viral replication [116]. The importance of organoid cultures in enteric virus research revealed multiple facets of gut-specific infections, regulating both the virus life cycle and host responses [135–139]. Most studies on these neurotropic enteroviruses have been on intestinal organoid models, but numerous neural models are available that can be useful for addressing CNS-related pathology [128]. Such neural organoids have been used for other viral infections to identify cell tropism and innate immune responses, observe relevant events such as microcephaly, and perform antiviral testing [140].

Although these initial studies highlight the utility of stem cell-derived models for studying neurotropic enteric virus pathogenesis along the gut-brain axis, they have limitations that should be considered. Primary cell-derived epithelial organoid models only contain a single cell layer. For instance, intestinal enteroids only include (specific) epithelial cells and do not include mesenchymal, immune, or nerve cells [141]. On the other hand, pluripotent stem cell-derived organoids, such as neural organoids, only represent certain embryonic brain regions [141]. Furthermore, as a nascent technology, the use of different protocols influences the robustness of these models, and these differences should be considered when interpreting results [142]. However, many of these limitations continue to be addressed, and increasing the complexity of human stem cell-derived systems holds promise for a better understanding of the detailed functions of neurotropic enteroviruses along this axis.

### Complex engineered systems

The combination of advances in engineering and biology enables the development of complex systems, such as organ-on-chip models that incorporate both physiological complexity and cellular diversity, driving viral dissemination [143]. Progress has been made in recent years to integrate physiological hallmarks such as spatial arrangement and perfusion in an intestinal organoid-on-chip system for studying host-microorganism interactions [144]. These stem cell-derived systems also allow exploring the role of intestinal commensals and their influence on pathogens [145]. Regarding the nervous system, assembloids resulting from the fusion of different regionalized neural organoids can model human-specific aspects of neural circuitry [146, 147]. Ultimately, stem cell-derived multi-organ-on-chip models, in combination with next-generation sequencing, integrative omics, and machine learning, can pave the way for delineating host and viral factors that drive enteric pathogens to become neurotropic.

## Conclusions and outstanding questions

This review provides an overview of neurotropic enteric viruses and host complexity along the gut-brain axis. We also speculated on the possibility of the intestinal tract as a site for selection due to its unique properties. Some outstanding questions on neurotropic enteric viruses and their transition from the gut to the brain are listed in *box 2* to stimulate further discussion. We focused our review on two viral families associated with CNS pathology following enteric infection. However, studies now suggest that other common intestinal viruses, such as noroviruses and reoviruses, may also act along this axis [5, 148]. Furthermore, classical neurotropic viruses like CMV can also, in specific instances such as postnatal infection in preterm infants, use the intestinal tract as the primary entry site [149, 150]. Thus, there is increasing evidence to suspect the gut-brain axis is a major route for neurotropic viruses, a topic that is underappreciated and warrants further research.

**Table:** 

Box 2 Outstanding questions in the field
Which RNA elements represent common virulence factors and may be used as therapeutic targets?
Can the CNS pathology be attributed to similar RNA elements between different virus groups? Can these be predicted using AI algorithms?
How are virus and host translation regulated in gut vs CNS cells?
How important are immune responses for CNS pathology and can they be manipulated therapeutically?
What is the weakest link (bottleneck, barrier) for the virus in the gut-brain axis?
What is the likelihood of CNS infections? Do chronic intestinal infections play a role in CNS dissemination?
Are there links of intestinal viral infections and neurological disorders, type I diabetes, and other long-term conditions?
How are the routes to other tissues such as liver (E11) and cardiovascular system (CVB5) regulated?
What is the role of the gut microbiome in virus movement along the gut-brain axis?

## Data Availability

Not applicable.

## Abbreviations

AFP: Acute flaccid paralysis
BBB: Blood-brain barrier
BCSFB: Blood-cerebrospinal fluid barrier
CNS: Central nervous system
CSF: Cerebrospinal fluid
EEC: Enteroendocrine cells
ENS: Enteric nervous system
HFMD: Hand, foot and mouth disease
IRES: Internal ribosome entry site
PNS: Peripheral nervous system

## References

[1] Lockhart A, Mucida D, Parsa R (2022) Immunity to enteric viruses. Immunity 55:800–818 35545029 10.1016/j.immuni.2022.04.007 PMC9257994

[2] Bernard-Raichon L, Cadwell K (2023) Immunomodulation by enteric viruses. Annu Rev Virol 10:477–502 37380186 10.1146/annurev-virology-111821-112317

[3] Blutt SE, Kirkwood CD, Parreño V, Warfield KL, Ciarlet M, Estes MK et al (2003) Rotavirus antigenaemia and viraemia: a common event? Lancet 362:1445–1449 14602437 10.1016/S0140-6736(03)14687-9

[4] Hellysaz A, Hagbom M (2021) Understanding the central nervous system symptoms of rotavirus: a qualitative review. Viruses. 10.3390/V13040658 33920421 10.3390/v13040658 PMC8069368

[5] Deb S, Mondal R, Lahiri D, Shome G, Roy AG, Sarkar V et al (2023) Norovirus-associated neurological manifestations: summarizing the evidence. J Neurovirol 29:492–506 37477790 10.1007/s13365-023-01152-0 PMC10501950

[6] Liu Y, Sheng J, van Vliet ALW, Buda G, van Kuppeveld FJM, Rossmann MG (2018) Molecular basis for the acid-initiated uncoating of human enterovirus D68. Proc Natl Acad Sci U S A 115:E12209–E12217 30530701 10.1073/pnas.1803347115 PMC6310856

[7] Chang LY, Lin HY, Gau SSF, Lu CY, Hsia SH, Huang YC et al (2019) Enterovirus A71 neurologic complications and long-term sequelae. J Biomed Sci. 10.1186/S12929-019-0552-7 31395054 10.1186/s12929-019-0552-7 PMC6688366

[8] Yip CCY, Lau SKP, Woo PCY, Yuen KY (2013) Human enterovirus 71 epidemics: what’s next? Emerg Health Threats J 6:19780 24119538 10.3402/ehtj.v6i0.19780 PMC3772321

[9] Li J, Wang X, Cai J, Ge Y, Wang C, Qiu Y et al (2020) Non-polio enterovirus infections in children with central nervous system disorders in Shanghai, 2016–2018: serotypes and clinical characteristics. J Clin Virol. 10.1016/J.JCV.2020.104516 32585621 10.1016/j.jcv.2020.104516

[10] Gonzalez G, Carr MJ, Kobayashi M, Hanaoka N, Fujimoto T (2019) Enterovirus-associated hand-foot and mouth disease and neurological complications in japan and the rest of the world. Int J Mol Sci 20:5201. 10.3390/IJMS20205201 31635198 10.3390/ijms20205201 PMC6834195

[11] Tao Z, Wang H, Liu Y, Li Y, Jiang P, Liu G et al (2014) Non-polio enteroviruses from acute flaccid paralysis surveillance in Shandong Province, China, 1988-2013. Sci Rep. 10.1038/SREP06167 25145609 10.1038/srep06167 PMC4141246

[12] Ndiaye N, Kébé O, Diarra M, Thiaw FD, Dia M, Dia NdD et al (2024) Non-polio enteroviruses circulation in acute flaccid paralysis cases and sewage in Senegal from 2013 to 2021. Int J Infect Dis 138:54–62 37995831 10.1016/j.ijid.2023.11.020

[13] Kohil A, Jemmieh S, Smatti MK, Yassine HM (2021) Viral meningitis: an overview. Arch Virol 166:335–345 33392820 10.1007/s00705-020-04891-1 PMC7779091

[14] Racaniello VR (2006) One hundred years of poliovirus pathogenesis. Virology 344:9–16 16364730 10.1016/j.virol.2005.09.015

[15] Ponomareva NV, Novikova NA (2023) Neurotropic enteroviruses (Picornaviridae: Enterovirus): predominant types, basis of neurovirulence. Vopr Virusol 68:479–487 38156564 10.36233/0507-4088-205

[16] Suresh S, Forgie S, Robinson J (2018) Non-polio enterovirus detection with acute flaccid paralysis: a systematic review. J Med Virol 90:3–7 28857219 10.1002/jmv.24933

[17] Tapparel C, Siegrist F, Petty TJ, Kaiser L (2013) Picornavirus and enterovirus diversity with associated human diseases. Infect Genet Evol 14:282–293 23201849 10.1016/j.meegid.2012.10.016

[18] Adeniji JA, Faleye TOC (2015) Enterovirus C strains circulating in Nigeria and their contribution to the emergence of recombinant circulating vaccine-derived polioviruses. Arch Virol 160:675–683 25559670 10.1007/s00705-014-2322-x

[19] Sridhar A, Karelehto E, Brouwer L, Pajkrt D, Wolthers KC (2019) Parechovirus A pathogenesis and the enigma of genotype A-3. Viruses 11:1062. 10.3390/V11111062 31739613 10.3390/v11111062 PMC6893760

[20] Bubba L, Broberg EK, Fischer TK, Simmonds P, Harvala H (2024) Parechovirus A circulation and testing capacities in Europe, 2015–2021. Emerg Infect Dis 30:234–244 38270192 10.3201/eid3002.230647 PMC10826775

[21] Bosch A, Pintó RM, Guix S (2014) Human astroviruses. Clin Microbiol Rev 27:1048–1074 25278582 10.1128/CMR.00013-14 PMC4187635

[22] Cortez V, Meliopoulos VA, Karlsson EA, Hargest V, Johnson C, Schultz-Cherry S (2017) Astrovirus biology and pathogenesis. Annu Rev Virol 4:327–348 28715976 10.1146/annurev-virology-101416-041742

[23] Farahmand M, Khales P, Salavatiha Z, Sabaei M, Hamidzade M, Aminpanah D et al (2023) Worldwide prevalence and genotype distribution of human astrovirus in gastroenteritis patients: a systematic review and meta-analysis. Microb Pathog 181:106209. 10.1016/J.MICPATH.2023.106209 37385570 10.1016/j.micpath.2023.106209

[24] Zhu P, Ji W, Li D, Li Z, Chen Y, Dai B et al (2023) Current status of hand-foot-and-mouth disease. J Biomed Sci. 10.1186/S12929-023-00908-4 36829162 10.1186/s12929-023-00908-4 PMC9951172

[25] Chandran D, Chakraborty S, Ahmed SK, Chopra H, Islam MR, Dhama K (2023) France reports rise in severe neonatal infections caused by a new enterovirus (Echovirus-11) variant. Clin Pathol. 10.1177/2632010X231213793 38022906 10.1177/2632010X231213793 PMC10657519

[26] Bello AM, Roshorm YM (2022) Recent progress and advances towards developing enterovirus 71 vaccines for effective protection against human hand, foot and mouth disease (HFMD). Biologicals 79:1–9 36089444 10.1016/j.biologicals.2022.08.007

[27] Chong P, Liu CC, Chow YH, Chou AH, Klein M (2015) Review of enterovirus 71 vaccines. Clin Infect Dis 60:797–803 25352588 10.1093/cid/ciu852

[28] Kasarello K, Cudnoch-Jedrzejewska A, Czarzasta K (2023) Communication of gut microbiota and brain via immune and neuroendocrine signaling. Front Microbiol. 10.3389/FMICB.2023.1118529 36760508 10.3389/fmicb.2023.1118529 PMC9907780

[29] Sharkey KA, Mawe GM (2023) The enteric nervous system. Physiol Rev 103:1487–1564 36521049 10.1152/physrev.00018.2022 PMC9970663

[30] Carloni S, Rescigno M (2022) Unveiling the gut-brain axis: structural and functional analogies between the gut and the choroid plexus vascular and immune barriers. Semin Immunopathol 44:869–882 35861857 10.1007/s00281-022-00955-3 PMC9301898

[31] Collins SM, Surette M, Bercik P (2012) The interplay between the intestinal microbiota and the brain. Nat Rev Microbiol 10:735–742 23000955 10.1038/nrmicro2876

[32] Arrieta MC, Stiemsma LT, Amenyogbe N, Brown E, Finlay B (2014) The intestinal microbiome in early life: health and disease. Front Immunol. 10.3389/FIMMU.2014.00427 25250028 10.3389/fimmu.2014.00427 PMC4155789

[33] Schächtle MA, Rosshart SP (2021) The microbiota-gut-brain axis in health and disease and its implications for translational research. Front Cell Neurosci. 10.3389/FNCEL.2021.698172 34335190 10.3389/fncel.2021.698172 PMC8321234

[34] Buffington SA, Di Prisco GV, Auchtung TA, Ajami NJ, Petrosino JF, Costa-Mattioli M (2016) Microbial reconstitution reverses maternal diet-induced social and synaptic deficits in offspring. Cell 165:1762–1775 27315483 10.1016/j.cell.2016.06.001 PMC5102250

[35] Hou K, Wu ZX, Chen XY, Wang JQ, Zhang D, Xiao C et al (2022) Microbiota in health and diseases. Signal Transduct Target Ther. 10.1038/S41392-022-00974-4 35461318 10.1038/s41392-022-00974-4 PMC9034083

[36] Latorre R, Sternini C, De Giorgio R, Greenwood-Van MB (2016) Enteroendocrine cells: a review of their role in brain-gut communication. Neurogastroenterol Motility 28:620–630 10.1111/nmo.12754 PMC4842178 26691223

[37] Al OY, Aziz Q (2014) The brain-gut axis in health and disease. Adv Exp Med Biol. 817:135–153 24997032 10.1007/978-1-4939-0897-4_6

[38] Lyte M (2013) Microbial endocrinology in the microbiome-gut-brain axis: how bacterial production and utilization of neurochemicals influence behavior. PLoS Pathog. 10.1371/JOURNAL.PPAT.1003726 24244158 10.1371/journal.ppat.1003726 PMC3828163

[39] Quigley EMM (2017) Microbiota-brain-gut axis and neurodegenerative diseases. Curr Neurol Neurosci Rep. 10.1007/S11910-017-0802-6 29039142 10.1007/s11910-017-0802-6

[40] Brun P, Qesari M, Marconi PC, Kotsafti A, Porzionato A, Macchi V et al (2018) Herpes simplex virus type 1 infects enteric neurons and triggers gut dysfunction via macrophage recruitment. Front Cell Infect Microbiol. 10.3389/FCIMB.2018.00074 29600197 10.3389/fcimb.2018.00074 PMC5862801

[41] Green KY, Kaufman SS, Nagata BM, Chaimongkol N, Kim DY, Levenson EA et al (2020) Human norovirus targets enteroendocrine epithelial cells in the small intestine. Nat Commun. 10.1038/S41467-020-16491-3 32488028 10.1038/s41467-020-16491-3 PMC7265440

[42] Kim AHJ, Hogarty MP, Harris VC, Baldridge MT (2021) The complex interactions between rotavirus and the gut microbiota. Front Cell Infect Microbiol. 10.3389/FCIMB.2020.586751 33489932 10.3389/fcimb.2020.586751 PMC7819889

[43] Magwira CA, Taylor MB (2018) Composition of gut microbiota and its influence on the immunogenicity of oral rotavirus vaccines. Vaccine 36:3427–3433 29752022 10.1016/j.vaccine.2018.04.091

[44] Guedes-Dias P, Holzbaur ELF (2019) Axonal transport: driving synaptic function. Science. 10.1126/SCIENCE.AAW9997 31601744 10.1126/science.aaw9997 PMC6996143

[45] Pegg CE, Zaichick SV, Bomba-Warczak E, Jovasevic V, Kim DH, Kharkwal H et al (2021) Herpesviruses assimilate kinesin to produce motorized viral particles. Nature 599:662–666 34789877 10.1038/s41586-021-04106-w PMC8675142

[46] Potratz M, Zaeck LM, Weigel C, Klein A, Freuling CM, Müller T et al (2020) Neuroglia infection by rabies virus after anterograde virus spread in peripheral neurons. Acta Neuropathol Commun. 10.1186/S40478-020-01074-6 33228789 10.1186/s40478-020-01074-6 PMC7684951

[47] Callaway EM (2008) Transneuronal circuit tracing with neurotropic viruses. Curr Opin Neurobiol 18:617–623 19349161 10.1016/j.conb.2009.03.007 PMC2698966

[48] Schiller M, Ben-Shaanan TL, Rolls A (2020) Neuronal regulation of immunity: why, how and where? Nat Rev Immunol 21:20–36 32811994 10.1038/s41577-020-0387-1

[49] Koyuncu OO, Hogue IB, Enquist LW (2013) Virus infections in the nervous system. Cell Host Microbe 13:379–393 23601101 10.1016/j.chom.2013.03.010 PMC3647473

[50] Enquist LW, Leib DA (2016) Intrinsic and innate defenses of neurons: détente with the herpesviruses. J Virol. 10.1128/JVI.01200-16 27795407 10.1128/JVI.01200-16 PMC5165195

[51] Nomoto A (2007) Molecular aspects of poliovirus pathogenesis. Proc Jpn Acad Ser B Phys Biol Sci 83:266–275 24367151 10.2183/pjab/83.266 PMC3859295

[52] Louveau A, Herz J, Alme MN, Salvador AF, Dong MQ, Viar KE et al (2018) CNS lymphatic drainage and neuroinflammation are regulated by meningeal lymphatic vasculature. Nat Neurosci 21:1380–1391 30224810 10.1038/s41593-018-0227-9 PMC6214619

[53] Licastro E, Pignataro G, Iliff JJ, Xiang Y, Lo EH, Hayakawa K et al (2024) Glymphatic and lymphatic communication with systemic responses during physiological and pathological conditions in the central nervous system. Commun Biol. 10.1038/S42003-024-05911-5 38402351 10.1038/s42003-024-05911-5 PMC10894274

[54] Solár P, Zamani A, Kubíčková L, Dubový P, Joukal M (2020) Choroid plexus and the blood-cerebrospinal fluid barrier in disease. Fluids Barriers CNS. 10.1186/S12987-020-00196-2 32375819 10.1186/s12987-020-00196-2 PMC7201396

[55] Redzic ZB, Segal MB (2004) The structure of the choroid plexus and the physiology of the choroid plexus epithelium. Adv Drug Deliv Rev 56:1695–1716 15381330 10.1016/j.addr.2004.07.005

[56] Spector R, Keep RF, Robert Snodgrass S, Smith QR, Johanson CE (2015) A balanced view of choroid plexus structure and function: focus on adult humans. Exp Neurol 267:78–86 25747036 10.1016/j.expneurol.2015.02.032

[57] Vigh-Teichmann I, Vigh B (1983) The system of cerebrospinal fluid-contacting neurons. Arch Histol Jpn 46:427–468 6362609 10.1679/aohc.46.427

[58] Kupila L, Vuorinen T, Vainionpää R, Marttila RJ, Kotilainon P (2005) Diagnosis of enteroviral meningitis by use of polymerase chain reaction of cerebrospinal fluid, stool, and serum specimens. Clin Infect Dis 40:982–987 15824990 10.1086/428581

[59] Langen UH, Ayloo S, Gu C (2019) Development and cell biology of the blood-brain barrier. Annu Rev Cell Dev Biol 35:591–613 31299172 10.1146/annurev-cellbio-100617-062608 PMC8934576

[60] Abbott NJ, Patabendige AAK, Dolman DEM, Yusof SR, Begley DJ (2010) Structure and function of the blood-brain barrier. Neurobiol Dis 37:13–25 19664713 10.1016/j.nbd.2009.07.030

[61] Wu D, Chen Q, Chen X, Han F, Chen Z, Wang Y (2023) The blood-brain barrier: structure, regulation, and drug delivery. Signal Transduct Target Ther. 10.1038/S41392-023-01481-W 37231000 10.1038/s41392-023-01481-w PMC10212980

[62] Kim KS (2008) Mechanisms of microbial traversal of the blood-brain barrier. Nat Rev Microbiol 6:625–634 18604221 10.1038/nrmicro1952 PMC5206914

[63] Horowitz A, Chanez-Paredes SD, Haest X, Turner JR (2023) Paracellular permeability and tight junction regulation in gut health and disease. Nat Rev Gastroenterol Hepatol 20:1 37186118 10.1038/s41575-023-00766-3 PMC10127193

[64] Hasebe R, Suzuki T, Makino Y, Igarashi M, Yamanouchi S, Maeda A et al (2010) Transcellular transport of West Nile virus-like particles across human endothelial cells depends on residues 156 and 159 of envelope protein. BMC Microbiol 10:165 20529314 10.1186/1471-2180-10-165 PMC2889955

[65] Gaume L, Chabrolles H, Bisseux M, Lopez-Coqueiro I, Dehouck L, Mirand A et al (2024) Enterovirus A71 crosses a human blood-brain barrier model through infected immune cells. Microbiol Spectr. 10.1128/SPECTRUM.00690-24 38752731 10.1128/spectrum.00690-24 PMC11237604

[66] Cheng HY, Huang YC, Yen TY, Hsia SH, Hsieh YC, Li CC et al (2014) The correlation between the presence of viremia and clinical severity in patients with enterovirus 71 infection: a multi-center cohort study. BMC Infect Dis. 10.1186/1471-2334-14-417 25069383 10.1186/1471-2334-14-417 PMC4133623

[67] Lin Y-W, Chang K-C, Kao C-M, Chang S-P, Tung Y-Y, Chen S-H (2009) Lymphocyte and antibody responses reduce enterovirus 71 lethality in mice by decreasing tissue viral loads. J Virol 83:6477–6483 19386699 10.1128/JVI.00434-09 PMC2698549

[68] Wagner JN, Leibetseder A, Troescher A, Panholzer J, von Oertzen TJ (2021) Characteristics and therapy of enteroviral encephalitis: case report and systematic literature review. Int J Infect Dis 113:93–102 34628025 10.1016/j.ijid.2021.10.002

[69] Helander HF, Fändriks L (2014) Surface area of the digestive tract—revisited. Scand J Gastroenterol 49:681–689 24694282 10.3109/00365521.2014.898326

[70] Turnbaugh PJ, Ley RE, Hamady M, Fraser-Liggett CM, Knight R, Gordon JI (2007) The human microbiome project. Nature 449:804–810 17943116 10.1038/nature06244 PMC3709439

[71] Peña-gil N, Santiso-bellón C, Gozalbo-rovira R, Buesa J, Monedero V, Rodríguez-díaz J (2021) The role of host glycobiology and gut microbiota in rotavirus and norovirus infection, an update. Int J Mol Sci. 10.3390/IJMS222413473 34948268 10.3390/ijms222413473 PMC8704558

[72] Robinson CM, Jesudhasan PR, Pfeiffer JK (2014) Bacterial lipopolysaccharide binding enhances virion stability and promotes environmental fitness of an enteric virus. Cell Host Microbe 15:36–46 24439896 10.1016/j.chom.2013.12.004 PMC3920179

[73] Robinson CM, Woods Acevedo MA, McCune BT, Pfeiffer JK (2019) Related enteric viruses have different requirements for host microbiota in mice. J Virol. 10.1128/JVI.01339-19 31511379 10.1128/JVI.01339-19 PMC6854509

[74] Kuss SK, Best GT, Etheredge CA, Pruijssers AJ, Frierson JM, Hooper LV et al (2011) Intestinal microbiota promote enteric virus replication and systemic pathogenesis. Science 334:249–252 21998395 10.1126/science.1211057 PMC3222156

[75] Ma C, Wu X, Nawaz M, Li J, Yu P, Moore JE et al (2011) Molecular characterization of fecal microbiota in patients with viral diarrhea. Curr Microbiol 63:259–266 21739252 10.1007/s00284-011-9972-7

[76] Cortez V, Margolis E, Schultz-Cherry S (2019) Astrovirus and the microbiome. Curr Opin Virol 37:10–15 31163291 10.1016/j.coviro.2019.05.002 PMC6768711

[77] Fleming MA, Ehsan L, Moore SR, Levin DE (2020) The enteric nervous system and its emerging role as a therapeutic target. Gastroenterol Res Pract. 10.1155/2020/8024171 32963521 10.1155/2020/8024171 PMC7495222

[78] Al OY, Aziz Q (2014) The brain-gut axis in health and disease. Adv Exp Med Biol. 817:135–153 24997032 10.1007/978-1-4939-0897-4_6

[79] Granger DN, Holm L, Kvietys P (2015) The gastrointestinal circulation: physiology and pathophysiology. Compr Physiol 5:1541–1583 26140727 10.1002/cphy.c150007

[80] Kuss SK, Etheredge CA, Pfeiffer JK (2008) Multiple host barriers restrict poliovirus trafficking in mice. PLoS Pathog. 10.1371/JOURNAL.PPAT.1000082 18535656 10.1371/journal.ppat.1000082 PMC2390757

[81] Tseligka ED, Sobo K, Stoppini L, Cagno V, Abdul F, Piuz I et al (2018) A VP1 mutation acquired during an enterovirus 71 disseminated infection confers heparan sulfate binding ability and modulates ex vivo tropism. PLoS Pathog. 10.1371/JOURNAL.PPAT.1007190 30075025 10.1371/journal.ppat.1007190 PMC6093697

[82] Saunderson R, Yu B, Trent RJ, Pamphlett R (2004) A polymorphism in the poliovirus receptor gene differs in motor neuron disease. Neuroreport 15:383–386 15076773 10.1097/00001756-200402090-00034

[83] Vignuzzi M, Stone JK, Arnold JJ, Cameron CE, Andino R (2006) Quasispecies diversity determines pathogenesis through cooperative interactions in a viral population. Nature 439:344–348 16327776 10.1038/nature04388 PMC1569948

[84] Pfeiffer JK (2010) Innate host barriers to viral trafficking and population diversity: lessons learned from poliovirus. Adv Virus Res 77:85–118 20951871 10.1016/B978-0-12-385034-8.00004-1 PMC3234684

[85] Ali H, Lulla A, Nicholson AS, Hankinson J, Wignall-Fleming EB, Connor RLO et al (2023) Attenuation hotspots in neurotropic human astroviruses. PLoS Biol. 10.1371/JOURNAL.PBIO.3001815 37459343 10.1371/journal.pbio.3001815 PMC10374088

[86] Racaniello VR, Baltimore D (1981) Molecular cloning of poliovirus cDNA and determination of the complete nucleotide sequence of the viral genome. Proc Natl Acad Sci USA 78:4887–91 6272282 10.1073/pnas.78.8.4887 PMC320284

[87] Jia QJ, Chen XY, Li DZ, Xu JJ, Xu ZG, Duan ZL et al (2016) Comparative genomic analysis of enterovirus 71 revealed six new potential neurovirulence-associated sites. Biomed Environ Sci 29:767–772 27927278 10.3967/bes2016.103

[88] Sonenberg N, Hinnebusch AG (2009) Regulation of translation initiation in eukaryotes: mechanisms and biological targets. Cell 136:731–745 19239892 10.1016/j.cell.2009.01.042 PMC3610329

[89] La Monica N, Racaniello VR (1989) Differences in replication of attenuated and neurovirulent polioviruses in human neuroblastoma cell line SH-SY5Y. J Virol 63:2357–2360 2539524 10.1128/jvi.63.5.2357-2360.1989 PMC250657

[90] Haller AA, Stewart SR, Semler BL (1996) Attenuation stem-loop lesions in the 5’ noncoding region of poliovirus RNA: neuronal cell-specific translation defects. J Virol 70:1467–1474 8627664 10.1128/jvi.70.3.1467-1474.1996 PMC189967

[91] Gutiérrez AL, Denova-Ocampo M, Racaniello VR, del Angel RM (1997) Attenuating mutations in the poliovirus 5’ untranslated region alter its interaction with polypyrimidine tract-binding protein. J Virol 71:3826–3833 9094658 10.1128/jvi.71.5.3826-3833.1997 PMC191533

[92] Kauder SE, Racaniello VR (2004) Poliovirus tropism and attenuation are determined after internal ribosome entry. J Clin Invest 113:1743–1753 15199409 10.1172/JCI21323 PMC420511

[93] Semler BL (2004) Poliovirus proves IRES-istible in vivo. J Clin Invest 113:1678–1681 15199401 10.1172/JCI22139 PMC420512

[94] Svitkin YV, Maslova SV, Agol VI (1985) The genomes of attenuated and virulent poliovirus strains differ in their in vitro translation efficiencies. Virology 147:243–252 3000069 10.1016/0042-6822(85)90127-8

[95] Chen L, Xu SJ, Yao XJ, Yang H, Zhang HL, Meng J et al (2020) Molecular epidemiology of enteroviruses associated with severe hand, foot and mouth disease in Shenzhen, China, 2014–2018. Arch Virol 165:2213–2227 32666145 10.1007/s00705-020-04734-z PMC7360124

[96] Wu G-H, Lee K-M, Kao C-Y, Shih S-R (2023) The internal ribosome entry site determines the neurotropic potential of enterovirus A71. Microbes Infect 25:105107 36708870 10.1016/j.micinf.2023.105107

[97] Lulla V, Dinan AM, Hosmillo M, Chaudhry Y, Sherry L, Irigoyen N et al (2019) An upstream protein-coding region in enteroviruses modulates virus infection in gut epithelial cells. Nat Microbiol 4:280–292 30478287 10.1038/s41564-018-0297-1 PMC6443042

[98] Kobayashi K, Koike S (2020) Cellular receptors for enterovirus A71. J Biomed Sci. 10.1186/S12929-020-0615-9 31924205 10.1186/s12929-020-0615-9 PMC6954530

[99] Ang PY, Chong CWH, Alonso S (2021) Viral determinants that drive Enterovirus-A71 fitness and virulence. Emerg Microbes Infect 10:713–724 33745413 10.1080/22221751.2021.1906754 PMC8043536

[100] Yeo H, Chong CWH, Chen EW, Lim ZQ, Ng QY, Yan B et al (2022) A Single amino acid substitution in structural protein VP2 abrogates the neurotropism of enterovirus A-71 in mice. Front Microbiol. 10.3389/FMICB.2022.821976 35369482 10.3389/fmicb.2022.821976 PMC8969769

[101] Huang SW, Wang YF, Yu CK, Su IJ, Wang JR (2012) Mutations in VP2 and VP1 capsid proteins increase infectivity and mouse lethality of enterovirus 71 by virus binding and RNA accumulation enhancement. Virology 422:132–143 22078110 10.1016/j.virol.2011.10.015

[102] Baggen J, Thibaut HJ, Strating JRPM, Van Kuppeveld FJM (2018) The life cycle of non-polio enteroviruses and how to target it. Nat Rev Microbiol 16:368–381 29626210 10.1038/s41579-018-0005-4

[103] Pfeiffer JK, Kirkegaard K (2003) A single mutation in poliovirus RNA-dependent RNA polymerase confers resistance to mutagenic nucleotide analogs via increased fidelity. Proc Natl Acad Sci U S A 100:7289–7294 12754380 10.1073/pnas.1232294100 PMC165868

[104] Acevedo A, Brodsky L, Andino R (2014) Mutational and fitness landscapes of an RNA virus revealed through population sequencing. Nature 505:686–690 24284629 10.1038/nature12861 PMC4111796

[105] Te Yeh M, Bujaki E, Dolan PT, Smith M, Wahid R, Konz J et al (2020) Engineering the live-attenuated polio vaccine to prevent reversion to virulence. Cell Host Microbe 27:736-751.e8 32330425 10.1016/j.chom.2020.04.003 PMC7566161

[106] Pfeiffer JK, Kirkegaard K (2005) Increased fidelity reduces poliovirus fitness and virulence under selective pressure in mice. PLoS Pathog 1:0102–0110 10.1371/journal.ppat.0010011 PMC1250929 16220146

[107] Burns CC, Shaw J, Jorba J, Bukbuk D, Adu F, Gumede N et al (2013) Multiple independent emergences of type 2 vaccine-derived polioviruses during a large outbreak in Northern Nigeria. J Virol 87:4907–4922 23408630 10.1128/JVI.02954-12 PMC3624331

[108] Stern A, Te Yeh M, Zinger T, Smith M, Wright C, Ling G et al (2017) The evolutionary pathway to virulence of an RNA virus. Cell 169:35-46.e19 28340348 10.1016/j.cell.2017.03.013 PMC5787669

[109] Woodman A, Arnold JJ, Cameron CE, Evans DJ (2016) Biochemical and genetic analysis of the role of the viral polymerase in enterovirus recombination. Nucleic Acids Res 44:6883–6895 27317698 10.1093/nar/gkw567 PMC5001610

[110] Kim H, Ellis VD, Woodman A, Zhao Y, Arnold JJ, Cameron CE (2019) RNA-dependent rna polymerase speed and fidelity are not the only determinants of the mechanism or efficiency of recombination. Genes (Basel). 10.3390/GENES10120968 31775299 10.3390/genes10120968 PMC6947342

[111] Lim ZQ, Ng QY, Oo Y, Chu JJH, Ng SY, Sze SK et al (2021) Enterovirus-A71 exploits peripherin and Rac1 to invade the central nervous system. EMBO Rep. 10.15252/EMBR.202051777 33871166 10.15252/embr.202051777 PMC8183415

[112] Lin JY, Lin JY, Kuo RL, Huang HI (2024) Heterogeneous nuclear ribonucleoprotein A3 binds to the internal ribosomal entry site of enterovirus A71 and affects virus replication in neural cells. J Cell Biochem. 10.1002/JCB.30575 38720641 10.1002/jcb.30575

[113] Feuer R, Mena I, Pagarigan R, Slifka MK, Whitton JL (2002) Cell cycle status affects coxsackievirus replication, persistence, and reactivation in vitro. J Virol 76:4430–4440 11932410 10.1128/JVI.76.9.4430-4440.2002 PMC155066

[114] Lin JY, Huang HI (2020) Autophagy is induced and supports virus replication in Enterovirus A71-infected human primary neuronal cells. Sci Rep. 10.1038/S41598-020-71970-3 32943650 10.1038/s41598-020-71970-3 PMC7499237

[115] Too IHK, Yeo H, Sessions OM, Yan B, Libau EA, Howe JLC et al (2016) Enterovirus 71 infection of motor neuron-like NSC-34 cells undergoes a non-lytic exit pathway. Sci Rep. 10.1038/SREP36983 27849036 10.1038/srep36983 PMC5111112

[116] Capendale PE, García-Rodríguez I, Ambikan AT, Mulder LA, Depla JA, Freeze E et al (2024) Parechovirus infection in human brain organoids: host innate inflammatory response and not neuro-infectivity correlates to neurologic disease. Nat Commun. 10.1038/S41467-024-46634-9 38514653 10.1038/s41467-024-46634-9 PMC10958052

[117] Arita M, Nagata N, Iwata N, Ami Y, Suzaki Y, Mizuta K et al (2007) An attenuated strain of enterovirus 71 belonging to genotype a showed a broad spectrum of antigenicity with attenuated neurovirulence in cynomolgus monkeys. J Virol 81:9386–9395 17567701 10.1128/JVI.02856-06 PMC1951441

[118] VandeBerg JL, Williams-Blangero S (1997) Advantages and limitations of nonhuman primates as animal models in genetic research on complex diseases. J Med Primatol 26:113–119 9379477 10.1111/j.1600-0684.1997.tb00042.x

[119] Cauvin AJ, Peters C, Brennan F (2015) Advantages and limitations of commonly used nonhuman primate species in research and development of biopharmaceuticals. The nonhuman primate in nonclinical drug development and safety assessment 2015. pp 379-395.

[120] Zhang Y, Cui W, Liu L, Wang J, Zhao H, Liao Y et al (2011) Pathogenesis study of enterovirus 71 infection in rhesus monkeys. Lab Invest 91:1337–1350 21555996 10.1038/labinvest.2011.82

[121] Andino R, Kirkegaard K, MacAdam A, Racaniello VR, Rosenfeld AB (2023) The picornaviridae family: knowledge gaps, animal models, countermeasures, and prototype pathogens. J Infect Dis 228:S427–S445 37849401 10.1093/infdis/jiac426 PMC13381290

[122] Ren R, Costantini F, Gorgacz EJ, Lee JJ, Racaniello VR (1990) Transgenic mice expressing a human poliovirus receptor: a new model for poliomyelitis. Cell 63:353–362 2170026 10.1016/0092-8674(90)90168-e

[123] Morosky S, Wells AI, Lemon K, Evans AS, Schamus S, Bakkenist CJ et al (2019) The neonatal Fc receptor is a pan-echovirus receptor. Proc Natl Acad Sci U S A 116:3758–3763 30808762 10.1073/pnas.1817341116 PMC6397586

[124] Yang C-H, Liang C-T, Jiang S-T, Chen K-H, Yang C-C, Cheng M-L et al (2019) A novel murine model expressing a chimeric mSCARB2/hSCARB2 receptor is highly susceptible to oral infection with clinical isolates of enterovirus 71. J Virol. 10.1128/JVI.00183-19 30894476 10.1128/JVI.00183-19 PMC6532076

[125] Van Der Sanden SMG, Sachs N, Koekkoek SM, Koen G, Pajkrt D, Clevers H et al (2018) Enterovirus 71 infection of human airway organoids reveals VP1-145 as a viral infectivity determinant. Emerg Microbes Infect. 10.1038/S41426-018-0077-2 29743570 10.1038/s41426-018-0077-2 PMC5943241

[126] Aknouch I, García-Rodríguez I, Giugliano FP, Calitz C, Koen G, van Eijk H et al (2023) Amino acid variation at VP1-145 of enterovirus A71 determines the viral infectivity and receptor usage in a primary human intestinal model. Front Microbiol. 10.3389/FMICB.2023.1045587 37138595 10.3389/fmicb.2023.1045587 PMC10149690

[127] Sridhar A, Simmini S, Ribeiro CMS, Tapparel C, Evers MM, Pajkrt D et al (2020) A perspective on organoids for virology research. Viruses. 10.3390/V12111341 33238561 10.3390/v12111341 PMC7700289

[128] Mulder LA, Depla JA, Sridhar A, Wolthers K, Pajkrt D, de Vieira Sá R (2023) A beginner’s guide on the use of brain organoids for neuroscientists: a systematic review. Stem Cell Res Ther. 10.1186/S13287-023-03302-X 37061699 10.1186/s13287-023-03302-x PMC10105545

[129] Han Y, Yang L, Lacko LA, Chen S (2022) Human organoid models to study SARS-CoV-2 infection. Nat Methods. 10.1038/S41592-022-01453-Y 35396481 10.1038/s41592-022-01453-y

[130] Moreni G, van Eijk H, Koen G, Johannesson N, Calitz C, Benschop K et al (2023) Non-polio enterovirus C replicate in both airway and intestine organotypic cultures. Viruses. 10.3390/V15091823 37766230 10.3390/v15091823 PMC10537321

[131] Helgers LC, Bhoekhan MS, Pajkrt D, Wolthers KC, Geijtenbeek TBH, Sridhar A (2022) Human dendritic cells transmit enterovirus A71 via heparan sulfates to target cells independent of viral replication. Microbiol Spectr. 10.1128/SPECTRUM.02822-22 36222686 10.1128/spectrum.02822-22 PMC9769767

[132] Karelehto E, Cristella C, Yu X, Sridhar A, Hulsdouw R, de Haan K et al (2018) Polarized entry of human parechoviruses in the airway epithelium. Front Cell Infect Microbiol. 10.3389/FCIMB.2018.00294 30211126 10.3389/fcimb.2018.00294 PMC6119779

[133] García-Rodríguez I, van Eijk H, Koen G, Pajkrt D, Sridhar A, Wolthers KC (2021) Parechovirus A infection of the intestinal epithelium: differences between genotypes A1 and A3. Front Cell Infect Microbiol. 10.3389/FCIMB.2021.740662 34790587 10.3389/fcimb.2021.740662 PMC8591172

[134] Cordey S, Petty TJ, Schibler M, Martinez Y, Gerlach D, van Belle S et al (2012) Identification of site-specific adaptations conferring increased neural cell tropism during human enterovirus 71 infection. PLoS Pathog 8:19 10.1371/journal.ppat.1002826 PMC3406088 22910880

[135] Kolawole AO, Mirabelli C, Hill DR, Svoboda SA, Janowski AB, Passalacqua KD et al (2019) Astrovirus replication in human intestinal enteroids reveals multi-cellular tropism and an intricate host innate immune landscape. PLoS Pathog. 10.1371/journal.ppat.1008057 31671153 10.1371/journal.ppat.1008057 PMC6957189

[136] Ettayebi K, Crawford SE, Murakami K, Broughman JR, Karandikar U, Tenge VR et al (2016) Replication of human noroviruses in stem cell-derived human enteroids. Science 353:1387–1393 27562956 10.1126/science.aaf5211 PMC5305121

[137] Kolawole AO, Wobus CE (2020) Gastrointestinal organoid technology advances studies of enteric virus biology. PLoS Pathog. 10.1371/JOURNAL.PPAT.1008212 31999791 10.1371/journal.ppat.1008212 PMC6991956

[138] Drummond CG, Bolock AM, Ma C, Luke CJ, Good M, Coyne CB (2017) Enteroviruses infect human enteroids and induce antiviral signaling in a cell lineage-specific manner. Proc Natl Acad Sci 114:1672–1677 28137842 10.1073/pnas.1617363114 PMC5320971

[139] Wells AI, Coyne CB (2019) Enteroviruses: a gut-wrenching game of entry, detection, and evasion. Viruses. 10.3390/V11050460 31117206 10.3390/v11050460 PMC6563291

[140] Depla JA, Mulder LA, de Sá RV, Wartel M, Sridhar A, Evers MM et al (2022) Human brain organoids as models for central nervous system viral infection. Viruses. 10.3390/V14030634 35337041 10.3390/v14030634 PMC8948955

[141] Taelman J, Diaz M, Guiu J (2022) Human intestinal organoids: promise and challenge. Front Cell Dev Biol. 10.3389/FCELL.2022.854740 35359445 10.3389/fcell.2022.854740 PMC8962662

[142] Zhou C, Wu Y, Wang Z, Liu Y, Yu J, Wang W et al (2023) Standardization of organoid culture in cancer research. Cancer Med 12:14375–14386 37081739 10.1002/cam4.5943 PMC10358246

[143] Ingber DE (2022) Human organs-on-chips for disease modelling, drug development and personalized medicine. Nat Rev Genet 23:467–491 35338360 10.1038/s41576-022-00466-9 PMC8951665

[144] Nikolaev M, Mitrofanova O, Broguiere N, Geraldo S, Dutta D, Tabata Y et al (2020) Homeostatic mini-intestines through scaffold-guided organoid morphogenesis. Nature 585:574–578 32939089 10.1038/s41586-020-2724-8

[145] Nanlohy NM, Johannesson N, Wijnands L, Arroyo L, de Wit J, den Hartog G et al (2024) Exploring host-commensal-pathogen dynamics in cell line and organotypic human intestinal epithelial models. iScience. 10.1016/J.ISCI.2024.109771 38711444 10.1016/j.isci.2024.109771 PMC11070716

[146] Miura Y, Li MY, Revah O, Yoon SJ, Narazaki G, Pașca SP (2022) Engineering brain assembloids to interrogate human neural circuits. Nat Protoc 17:15–35 34992269 10.1038/s41596-021-00632-z

[147] Makrygianni EA, Chrousos GP (2021) From brain organoids to networking assembloids: implications for neuroendocrinology and stress medicine. Front Physiol. 10.3389/FPHYS.2021.621970 34177605 10.3389/fphys.2021.621970 PMC8222922

[148] Forrest JC, Dermody TS (2003) Reovirus receptors and pathogenesis. J Virol 77:9109–9115 12915527 10.1128/JVI.77.17.9109-9115.2003 PMC187431

[149] Goelz R, Hamprecht K, Klingel K, Poets CF (2016) Intestinal manifestations of postnatal and congenital cytomegalovirus infection in term and preterm infants. J Clin Virol 83:29–36 27529309 10.1016/j.jcv.2016.08.289

[150] Porta A, Avanzini A, Bellini M, Crossignani RM, Fiocchi S, Martinelli S et al (2016) Neonatal gastrointestinal involvement and congenital cytomegalovirus. Pediatr Med Chir 38:75–79 10.4081/pmc.2016.134 28009139

